# Iatrogenic tracheal rupture after extubation in the context of acute decompensated heart failure and cardiac device implantation: a case report

**DOI:** 10.1093/omcr/omab051

**Published:** 2021-07-21

**Authors:** Nika Kuridze, Kakhaber Etsadashvili, Eteri Minadze, Nani Gonjilashvili, Mikheil Tsverava

**Affiliations:** 1 Faculty of Clinical and Translational Medicine, Ivane Javakhishvili Tbilisi State University, Tbilisi, Georgia; 2 Department of Rhythmology, G. Chapidze Emergency Cardiology Center, Tbilisi, Georgia; 3 Department of Internal Medicine/Pulmonology, G. Chapidze Emergency Cardiology Center, Tbilisi, Georgia

## Abstract

Iatrogenic tracheal rupture is a life-threatening airway complication. It has a very low reported incidence and is more prevalent in women and patients over 50 years of age. The most frequent clinical manifestations of tracheal injury are subcutaneous emphysema and respiratory distress. We report a case of a 65-year-old woman with cardiac resynchronization therapy defibrillator implantation under general anesthesia. Shortly after extubation, dyspnea and subcutaneous emphysema appeared. The X-ray showed pneumomediastinum, pneumopericardium and pneumoperitoneum. The tracheal rupture was confirmed by bronchoscopy. After conservative treatment, the patient's well-being improved, and she was discharged from the hospital in a satisfactory condition.

## INTRODUCTION

Iatrogenic tracheal rupture is a life-threatening airway complication. Its real incidence is largely unknown. However, some authors reported it to be as low as 0.005% [1]. The causes of rupture are considered to be the interlacing of anatomical, mechanical and uncertain factors. A clinical diagnosis can be made when subcutaneous emphysema, respiratory failure and pneumomediastinum develop. A suspected tracheal rupture should be confirmed by bronchoscopy to clarify the location and length of rupture. A rapid diagnosis and treatment are essential to avoid mediastinitis and other above-mentioned complications [2]. Decisions regarding surgical or conservative treatment must be made based on patient’s general state, clinical, radiological and endoscopic data. Prognosis of tracheal rupture depends mainly on the patient’s underlying disease, cause of intubation and the severity of the intervention.

## CASE REPORT

**Figure 1:**
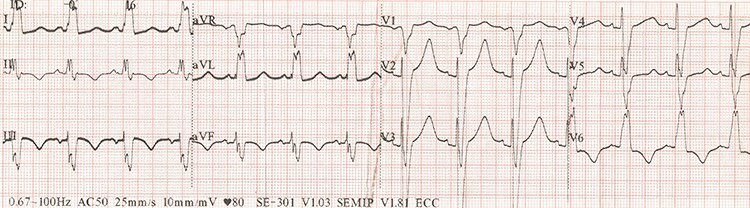
ECG before CRT-D implantation.

We report a case of a 65-year-old woman with a medical history of dilated cardiomyopathy, ventricular tachycardia, heart failure III-IV (NYHA), severe mitral insufficiency and severe pulmonary hypertension. Echocardiography shows left ventricular ejection fraction near 25%, biventricular dyssynchrony (septal to posterior wall motion delay 155 ms); on electrocardiogram—sinus rhythm, left bundle branch block, QRS—160 ms (Fig. 1). Surgical intervention of the mitral valve was not recommended by the cardiac surgeon due to the high risk to benefit ratio.

Despite the guideline-directed medical therapy with beta-blocker, ACE inhibitor, loop diuretic, mineralocorticoid receptor antagonist, the patient had frequent hospitalization due to the exacerbation of heart failure. Cardiac resynchronization therapy defibrillator (CRT-D) implantation was considered.

Due to the persistence of the acute heart failure symptoms despite the therapy with i/v loop diuretics, the CRT-D implantation was suggested as the only chance to improve the patient’s general condition. Since the patient was unable to maintain a supine position, the procedure was technically possible only under general anesthesia.

During the procedure, vital parameters were within the normal range. After the procedure, the patient was transferred to the intensive care unit for further observation. The patient was awakened and extubated after full assessment of her vital parameters. Within 10 minutes of her extubation, the patient’s condition became extremely critical. She started to suffer dyspnea, panting, shallow breathing, acrocyanosis and desaturation. Pulse oximetry was near 45%. Inspection revealed subcutaneous emphysema within her face and chest area. With auscultation of the lungs, reduced respiratory sounds were noticed on both sides. The patient was hemodynamically unstable. Against the backdrop of vital evidence tracheal reintubation was introduced and mechanical ventilation was started.

**Figure 2:**
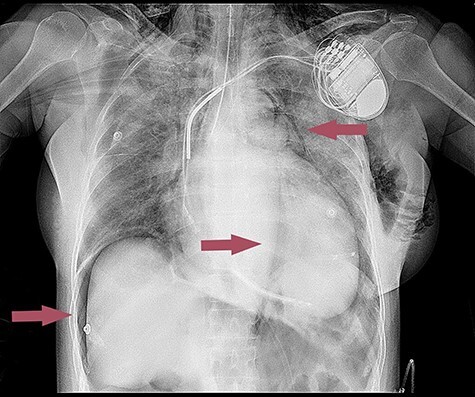
Chest X-ray after tracheal rupture.

Bronchoscopy confirmed rupture of the proximal part of the trachea, namely under the vocal cords. Chest X-ray showed subcutaneous emphysema, pneumopericardium, pneumomediastinum and signs of pneumoperitoneum in the right subdiaphragmatic space (Fig. 2). After reintubation patient’s respiratory symptoms were stable and per the thoracic surgeon’s decision patient remained under observation and conservative treatment, without further surgical intervention. Due to unstable hemodynamic parameters, which was caused by the decreased systolic function of the heart and rapid accumulation of the air in the pericardium, the patient received an intravenous infusion of dobutamine and norepinephrine. We observed an increase in inflammation markers such as C-reactive protein, erythrocyte sedimentation rate, white blood cells. The patient’s blood culture was inoculated, after which for the prevention of mediastinitis broad-spectrum antibiotic therapy (Ceftriaxone) was started, in addition to continued treatment for heart failure.

After symptoms were thus treated, the subcutaneous emphysema subsided, and the X-ray image revealed improvement. On the fourth day following the rupture incident, the patient was extubated successfully with bronchoscopic control, and her oxygen dependence was removed. The laboratory data reflected significant improvement, and the patient was discharged from the hospital with guideline-recommended medication for heart failure.

**Figure 3:**
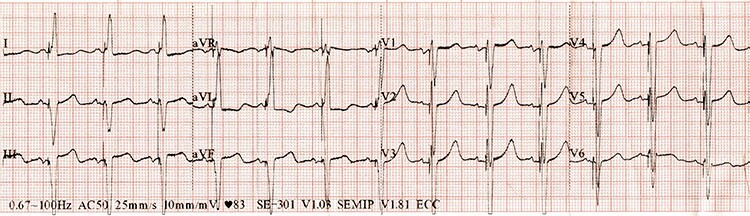
ECG after CRT-D implantation.

One month later, at a follow-up visit, the patient confirmed improvement in her well-being, heart failure symptoms had decreased (from NYHA III–IV to NYHA II–III), the ejection fraction increased (from 25% to 29%), and there was a strong improvement of septal to posterior wall motion delay (from 155 ms to 135 ms) and QRS duration (from 160 ms to 120 ms) (Fig. 3).

## DISCUSSION

Adequate treatment of tracheal rupture depends on the size of the lesion, clinical manifestation, underlying pathology, respiratory status and overall condition of the patient [3]. If these factors allow, this complication should be treated with the least invasive methods, hence reducing the need to perform surgery [4]. A small tear (about 1 cm in length), as long as there is no gross air leak nor respiratory failure, can be treated conservatively [5]. If the size of the tear exceeds 2 cm and any relatively large and progressive subcutaneous emphysema and/or respiratory distress are observed, the most effective treatment under such circumstances is early surgical intervention [6].

However, some authors reported the effectiveness of conservative treatment even with larger lesions [7]. Conservative management should be prioritized whenever possible—where a patient does not require mechanical ventilation or if it is possible without any loss of tidal volume, and when subcutaneous emphysema is mild and does not cause airway obstruction [8]. According to different authors, this choice is independent of the length of the lesion and includes lesions close to the carina [9].

Some authors recommend conservative nonoperative therapy as the optimal choice for patients who will require prolonged mechanical ventilation to treat their underlying respiratory status [10]. Surgical intervention is strongly recommended if the damage compromises the full thickness of the posterior membrane, and mediastinal structures protrude into the tracheal lumen.

## CONCLUSION

During trachea rupture, treatment should be tailored to the individual’s needs, which will depend on the lesion’s location, size and severity. Conservative treatment is recommended with small lacerations or patients with stable respiratory dynamics. In case of relatively large damage with an unstable patient, surgical intervention is the stronger suggestion.
